# DNA methylation and proteomic profiling of postmortem brain tissue reveals epigenetic dysregulation and inflammatory signaling in fragile X-associated tremor/ataxia syndrome

**DOI:** 10.3389/fnmol.2026.1932396

**Published:** 2026-10-13

**Authors:** Reymundo Lozano, Xiao Lin, Randi Hagerman, Verónica Martínez-Cerdeño, Dalila Pinto

**Affiliations:** 1 Department of Genetics and Genomic Sciences, Icahn School of Medicine at Mount Sinai, New York, NY, United States; 2 Department of Pediatrics, Icahn School of Medicine at Mount Sinai, New York, NY, United States; 3 Department of Artificial Intelligence and Human Health, Icahn School of Medicine at Mount Sinai, New York, NY, United States; 4 Department of Psychiatry, Icahn School of Medicine at Mount Sinai, New York, NY, United States; 5 Mindich Child Health and Development Institute, Icahn School of Medicine at Mount Sinai, New York, NY, United States; 6 Icahn Genomics Institute, Icahn School of Medicine at Mount Sinai, New York, NY, United States; 7 Friedman Brain Institute, Icahn School of Medicine at Mount Sinai, New York, NY, United States; 8 MIND Institute and Department of Pediatrics, University of California Davis Health, Davis, Sacramento, CA, United States; 9 Institute for Pediatric Regenerative Medicine, University of California Davis Health, Davis, Sacramento, CA, United States

**Keywords:** CYP2E1, DNA methylation, epigenetics, *FMR1*, FXTAS, inflammatory signaling, neurodegeneration, proteomics

## Abstract

**Background:**

Fragile X-associated tremor/ataxia syndrome (FXTAS) is a late-onset neurodegenerative disorder caused by a CGG repeat expansion in the premutation range of *FMR1*. The epigenetic landscape of the FXTAS brain remains largely uncharacterized. We performed genome-wide DNA methylation profiling of postmortem prefrontal cortex and targeted proteomic profiling to investigate molecular alterations associated with FXTAS and evaluate convergence on inflammatory signaling.

**Methods:**

DNA methylation was profiled in postmortem prefrontal cortex from 27 male FXTAS cases and 29 male controls using Illumina MethylationEPIC arrays. Surrogate variable analysis was used to account for latent sources of variation. Differentially methylated positions (DMPs) were defined as well as differentially methylated regions (DMRs). Exploratory functional analyses and comparison with published FXTAS transcriptomic and neurodegenerative-disease methylation datasets. Targeted proteomic profiling using the Olink Inflammation panel was performed in 9 male FXTAS cases and 12 male controls.

**Results:**

We identified 98 DMPs which mapped to 70 genes. Notable DMP-associated genes included *CYP2E1*, related to oxidative stress; *TRAF3*, *BATF*, *RCOR1*, and *MSI2*, with roles in immune and NF-κB-related signaling; and genes associated with myelination (*LINGO1*), synaptic function (*SYT3*), and zinc homeostasis (*SLC39A4*). *TRAF3*, a negative regulator of NF-κB and type I interferon signaling, contained one of the most significant DMPs. Exploratory Reactome analysis highlighted biological themes related to inflammatory resolution, axonal growth inhibition, zinc homeostasis, and *CYP2E1* metabolism, although no pathway remained significant after multiple-testing correction. Transcription-factor motif enrichment and protein–protein interaction analyses identified no significant higher-order regulatory or interaction modules. Seven DMP-associated genes overlapped with differentially expressed genes from an independent FXTAS mouse transcriptomic study, whereas no gene-level overlap was detected with the Alzheimer’s, Parkinson’s, or Huntington’s disease methylation signatures examined. Olink profiling identified 60 significantly altered inflammation-related proteins, 59 of which showed lower abundance in FXTAS, including TLR3, IRAK1, IRAK4, IKBKG/NEMO, and C1QA.

**Conclusion:**

These findings identify previously unrecognized DNA methylation alterations in FXTAS prefrontal cortex involving genes related to oxidative stress, myelination, zinc homeostasis, and innate immune/NF-κB signaling. Convergence between methylation changes in immune-regulatory genes, including *TRAF3*, and a predominantly reduced inflammatory protein signature provides complementary evidence for altered inflammatory signaling in FXTAS.

## 1 Introduction

Fragile X-associated tremor/ataxia syndrome (FXTAS) is a late-onset, progressive neurodegenerative disorder caused by premutation alleles (55–200 CGG repeats) in the fragile X messenger ribonucleoprotein 1 gene (*FMR1*) (Hagerman et al., 2017; Hagerman and Hagerman, 2016) FXTAS predominantly affects males, with an estimated penetrance of approximately 40% among male premutation carriers older than 50 years and up to 75% among those aged 80 years or older. (Jacquemont et al., 2004) Clinically, FXTAS is characterized by intention tremor, cerebellar ataxia, cognitive decline, and neuropsychiatric manifestations. The neuropathological hallmark is the widespread presence of ubiquitin-positive intranuclear inclusions in neurons, oligodendrocytes, and astrocytes throughout the brain (Greco et al., 2002; Iwahashi et al., 2006; Ma et al., 2019; McLennan et al., 2026). The canonical pathogenic mechanism in FXTAS involves a toxic gain-of-function arising from the 2- to 8-fold elevation in *FMR1* mRNA levels in premutation carriers (Tassone et al., 2000). In FXTAS, the premutation allele retains transcriptional activity, producing CGG repeat-containing mRNA that sequesters RNA-binding proteins (RBPs) and undergoes repeat-associated non-AUG (RAN) translation to produce toxic polyglycine-containing proteins (Todd et al., 2013; Sellier et al., 2013).

Epigenetic modifications, particularly DNA methylation at cytosine-guanine dinucleotide (CpG) sites, are increasingly recognized as important regulators of neurodegeneration (Klein and De Jager, 2016). However, the broader epigenetic landscape of the FXTAS brain remains poorly characterized. Prior methylation studies in FXTAS have largely focused on blood-derived DNA or examined the *FMR1* locus in isolation (Godler et al., 2011; Shelton et al., 2016). Genome-wide DNA methylation arrays provide broad coverage of CpG sites across regulatory and genic regions. The Olink proximity extension assay (Assarsson et al., 2014) enables sensitive multiplex quantification of inflammation-related proteins, including cytokines, chemokines, immune receptors, complement-related proteins, and other inflammatory mediators. Rigorous preprocessing and statistical adjustment are essential in these studies, because technical artifacts and latent sources of biological and technical variation can otherwise confound molecular associations (Zhou et al., 2018). Surrogate variable analysis (SVA) provides a framework for estimating and adjusting for such unmeasured sources of variation in high-dimensional molecular data (Leek and Storey, 2007).

In this study, we performed genome-wide DNA methylation profiling and targeted inflammatory proteomics in postmortem prefrontal cortex (Brodmann area 9) from male FXTAS cases and male controls. We aimed to: (1) characterize the genome-wide methylation landscape of the FXTAS brain; (2) identify differentially methylated positions (DMPs) and their associated genes; (3) explore biological processes represented among the DMP-associated genes through pathway and gene-set analyses; (4) integrate methylation findings with targeted inflammatory proteomic profiling to identify convergent molecular signatures; and (5) formulate mechanistic hypotheses linking our findings to known FXTAS pathophysiology. Together, our findings reveal novel, previously unrecognized epigenetic alterations involving genes associated with innate immune/NF-κB signaling, oxidative stress, myelination, and zinc homeostasis.

## 2 Methods

### 2.1 Brain tissue samples

This study utilized postmortem brain tissue from 27 males with FXTAS and 29 male controls. All samples were obtained from the prefrontal cortex (Brodmann area 9). FXTAS cases were sourced from the UC Davis FXS/FXTAS Brain Repository. All donors had provided written informed consent for brain autopsy and the use of brain tissue and clinical information for research purposes. Diagnosis of FXTAS was confirmed based on the presence of the *FMR1* premutation allele, clinical features consistent with FXTAS, and/or the identification of ubiquitin-positive intranuclear inclusions on postmortem neuropathological examination.

Control brain tissues, free of significant neurological history and without *FMR1* premutation, were obtained from the Neuropathology Brain Bank and Research CoRE (NPBB) at Mount Sinai and from the UC Davis Brain Bank. Female cases were not included in this study to eliminate confounding effects of X-chromosome inactivation on methylation at the *FMR1* locus and throughout the genome. Demographic characteristics are summarized in Table 1.

**Table 1:** Demographic characteristics of postmortem brain tissue donors.

Characteristic	FXTAS	Controls	Wilcoxon rank-sum test *p*-value
Sex, *n*	Male, *n* = 27	Male, *n* = 29	–
Brain region	Prefrontal cortex, BA9	Prefrontal cortex, BA9	–
Age at death, years—mean ± SD	75.7 ± 7.9	69.7 ± 5.3	*p* = 0.058
Age at death, range in years	42–97	57–89	–
PMI, hours—mean ± SD	12.4 ± 8.9	25.1 ± 16.6	*p* = 0.053
Brain source repository	UC Davis	NPBB/UC Davis	–
Methylation Array platform	EPICv1: *n* = 10; EPICv2: *n* = 17	EPICv1: *n* = 10; EPICv2: *n* = 19	–

PMI, postmortem interval; NPBB, Neuropathology Brain Bank and Research CoRE (NPBB) at Icahn School of Medicine at Mount Sinai; BA9, Brodmann area 9.

### 2.2 Statistical analysis and visualization

Group differences in age and postmortem interval (PMI) were assessed by Wilcoxon rank-sum test. All statistical analyses and data visualizations were performed in R (v4.3.2). Heatmaps were generated using the pheatmap package (v1.0.8) with default complete linkage hierarchical clustering if not specified.

### 2.3 DNA extraction and bisulfite conversion

Genomic DNA was isolated from approximately 250 mg of prefrontal cortex tissue using the AllPrep DNA/RNA Mini Kit (Qiagen, Valencia, CA, USA) following the manufacturer’s protocol. DNA quantity was assessed by Qubit 2.0 Fluorometer (ThermoFisher Scientific, Waltham, MA, USA) and DNA quality was evaluated by agarose gel electrophoresis. Bisulfite conversion of 500 ng genomic DNA per sample was performed using the EZ DNA Methylation Kit (Zymo Research, Irvine, CA, USA).

### 2.4 DNA methylation array

Genome-wide DNA methylation profiling was performed using two versions of the Illumina Infinium MethylationEPIC BeadChip array: EPICv1 (850 k probes, 10 FXTAS and 10 controls) and EPICv2 (900 k probes, 17 FXTAS and 19 controls). Array hybridization and scanning were performed according to the manufacturer’s standard protocols at the Icahn School of Medicine at Mount Sinai Microarray Core Facility. One FXTAS tissue sample was assayed on both platforms and served as an internal technical replicate for cross-platform validation.

### 2.5 Methylation array data processing and quality control

Raw IDAT files were processed using the SeSAMe package (v1.20.0) in R (v4.3.2) (Zhou et al., 2018). The openSesame function was applied with background subtraction, non-linear dye bias correction, and calculation of methylation beta (*β*) values for each probe. For EPICv2 data, probes were collapsed to the common prefix (collapseToPfx = TRUE). The EPICv1 and EPICv2 beta-value matrices were merged by retaining only probes present on both platforms and whose IDs start with cg, yielding 718,960 CpG probes for downstream analyses. Probes with any missing beta values across samples were excluded prior to downstream analysis. Principal component analysis (PCA) was performed on the merged, corrected beta-value matrix to assess sample clustering by diagnosis, platform, and other covariates. Surrogate variable analysis (SVA) was performed using the sva R package (Leek et al., 2012) to identify latent sources of variation not associated with the disease condition.

### 2.6 Differential methylation analyses

Differential methylation between FXTAS cases and controls was assessed at the individual CpG-probe level (DMP) using the DML function within the SeSAMe framework, with FXTAS diagnosis as the primary variable of interest and five surrogate variables as covariates. Disease status was included as a protected variable during surrogate variable estimation to preserve disease-associated methylation variation during adjustment. Estimated effect sizes (Δ*β*, defined as the difference in methylation beta values between FXTAS and controls) and corresponding *p* values were extracted using the summaryExtractTest function. Because diagnosis comprised only two groups (FXTAS and control), the raw and F-statistic-based *p* values were equivalent. Besides DMPs, differentially methylated regions (DMRs) were additionally identified using the SeSAMe DMR function, which groups adjacent CpG probes showing concordant differential methylation into candidate regions. For each DMR, the number of supporting CpG probes and associated gene annotations were recorded.

As a sensitivity analysis, *β* values were converted to *M* values using the BetaValueToMValue function, and SVA and differential methylation analyses were repeated using *M* values (Du et al., 2010). Results from the *β*-value and M-value analyses were compared using Pearson correlation and linear regression of effect estimates and adjusted *p* values, as well as evaluating overlap among identified DMPs. Multiple-testing correction was applied using the Benjamini-Hochberg procedure (FDR). DMPs were defined as CpG probes meeting both an absolute methylation difference of |Δ*β*| > 0.10 and a Benjamini–Hochberg-adjusted *p* value < 0.05.

### 2.7 Gene annotation, and analysis of gene pathways, transcription-factor motifs, and protein–protein interaction networks

DMPs were annotated to genes using annotatr (v1.32.0) (Cavalcante and Sartor, 2017), with gene assignments cross-referenced against GENCODE v41 annotations (Frankish et al., 2019). Genomic features were prioritized in the following order: (1) promoter, (2) 1–5 kb upstream, (3) first exon, (4) 5’ UTR, (5) coding sequence (CDS), (6) exon, (7) exon-intron boundary, (8) intron-exon boundary, (9) intron, and (10) intergenic. The highest-priority associated gene(s) were retained for each DMP. Multiple genes were allowed when they shared the same annotation rank. Each DMP was also annotated according to CpG-island context as CpG island: north or south shore, north or south shelf, or open sea.

Pathway enrichment analysis was performed using the Reactome 2024 database (Milacic et al., 2024), with all 70 DMP-associated genes as the query set and default overrepresentation/projection settings against the full human Reactome pathway hierarchy. Multiple-testing correction was performed using FDR procedure. Pathway enrichment was evaluated based on overlap between the input gene set and Reactome-annotated pathways. The DMP-associated genes were also analyzed using g:Profiler (g:GOSt). Multiple-testing correction used the g:SCS method, which accounts for the hierarchical and overlapping structure of ontology terms; terms with corrected *p* < 0.05 were considered significant.

Transcription-factor binding-site enrichment was assessed in 100-bp flanking regions around DMPs using HOMER (findMotifsGenome.pl). The full set of CpG probes tested on the merged EPIC array was used as the background rather than the whole genome. Enrichment of motifs from HOMER’s curated vertebrate known-motif library was evaluated using a cumulative hypergeometric test with Benjamini–Hochberg correction. The analysis was repeated using the JASPAR vertebrate CORE collection of position frequency matrices. JASPAR profiles were converted to HOMER motif format and supplied to findMotifsGenome.pl. using -mknown, with the same target intervals, probe-derived background, and statistical procedure to allow direct comparison between motif libraries. Motifs with a Benjamini-adjusted *q*-value < 0.05 were considered significantly enriched. Protein–protein interaction networks were constructed from the DMP-associated genes using STRING (https://string-db.org/, v12.0) with default settings.

Overlap between the DMP-associated gene set and external gene lists was also assessed. External comparison sets included: (i) differentially expressed genes from a mouse multi-omic FXTAS study (Jin et al., 2026), and published brain DNA methylation signatures for (ii) Parkinson’s disease (Harvey et al., 2025; cross-cortical meta-analysis of 1,239 samples from 855 donors), (iii) Alzheimer’s disease (Smith et al., 2021; cross-cortical meta-analysis of 1,408 donors) (Smith et al., 2021), and (iv) Huntington’s disease (Wheildon et al., 2025; 38 entorhinal cortex and 37 striatal samples).

### 2.8 Proteomic profiling (Olink)

To evaluate protein-level alterations in inflammatory pathways and their convergence with the methylation findings, targeted proteomic profiling was performed on prefrontal cortex (BA9) tissue from a subset of the cohort using the Olink Inflammation panel. Profiling was performed by GENEWIZ, an Azenta Life Sciences company. The panel quantifies inflammation-related proteins using the Proximity Extension Assay (PEA) (Assarsson et al., 2014). After quality control, 366 proteins were retained for analysis. To maintain consistency with the methylation analysis, the proteomic comparison was restricted to males with a confirmed FXTAS or control diagnosis, comprising 9 FXTAS cases and 12 controls.

Surrogate variable analysis (SVA) was applied to the NPX matrix to estimate and adjust for latent sources of variation, using an approach analogous to that applied to the methylation data. Surrogate variables were estimated from residuals of a model containing diagnosis, and the number of significant SVs was determined by a permutation analysis (two significant SVs were retained). Differential protein abundance between FXTAS and controls was assessed for each protein by linear regression on NPX values, with diagnosis as the primary predictor and the two SVs included as covariates. *p* values were adjusted for multiple testing using the FDR procedure, and proteins with adjusted *p* < 0.05 were considered significant. Principal component analysis and a heatmap of significant proteins were generated from SV-adjusted NPX values, with hierarchical clustering applied to proteins. Finally, the FXTAS protein signature was compared with the previously published mass spectrometry-based proteomic study of FXTAS postmortem cortex (Holm et al., 2020), and the directional concordance was assessed among the proteins measured in both datasets.

## 3 Results

### 3.1 Study cohort

The study cohort comprised 27 male FXTAS cases and 29 male controls, all with prefrontal cortex (BA9) tissue profiled on the MethylationEPIC array. The FXTAS group had a mean age of 75.7 ± 7.9 years, and controls had a mean age of 69.7 ± 5.3 years. There was no significant difference in age between the two groups based on the Wilcoxon rank-sum test (*p* = 0.058). Mean PMI was 12.4 ± 8.9 h for FXTAS cases compared to 25.1 ± 16.6 h for controls (Wilcoxon rank-sum test, *p* = 0.053). Samples were distributed across EPICv1 (10 FXTAS, 10 controls) and EPICv2 (17 FXTAS, 19 controls) platforms (Table 1).

### 3.2 Genome-wide methylation landscape in FXTAS

After quality control and harmonization of the EPICv1 and EPICv2 datasets, 718,960 probes shared across both platforms were retained for analysis. PCA of SVA-adjusted methylation data showed partial separation of FXTAS and control samples, primarily along PC1, which explained 7.2% of the variance, while PC2 explained 5.4% (Figure 1A). Coloring the same PCA by EPIC array platform showed no evident platform-driven clustering. The modest variance explained by the leading PCs is consistent with the heterogeneous sources of variation expected in bulk postmortem brain tissue. Supplementary Figure S1C summarizes associations among the estimated surrogate variables (SVs) and known biological and technical covariates, including age, postmortem interval (PMI), diagnosis, and EPIC array platform. Several SVs were strongly associated with technical variation, particularly array platform, whereas diagnosis showed limited association with the estimated SVs.

**Figure 1:**
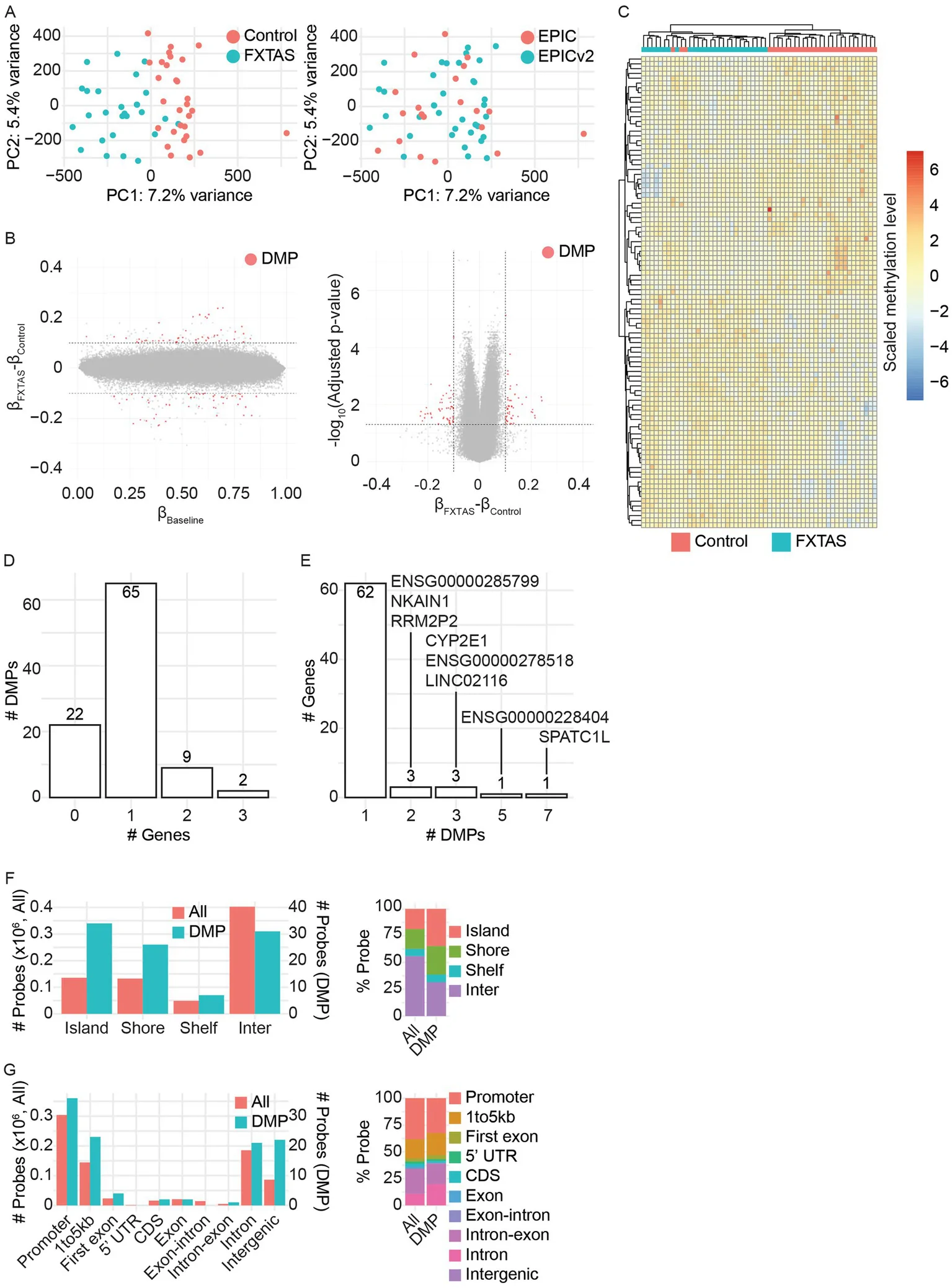
Genome-wide DNA methylation analysis of postmortem prefrontal cortex in FXTAS. **(A)** Principal component analysis (PCA) of SVA-adjusted methylation beta values from 27 male FXTAS cases and 29 male controls. Samples are displayed using identical PC coordinates and colored by diagnostic group (left; control, salmon; FXTAS, teal) or array platform (right; EPIC v1, salmon; EPIC v2, teal). *PC1* and *PC2* explain 7.2 and 5.4% of the variance, respectively. Partial separation by diagnostic group is observed, without evident clustering by array platform. **(C)** Heatmap of scaled methylation levels at the 98 significant DMPs (|*Δβ*| > 0.10, FDR < 0.05) across all 56 samples. Columns represent samples and rows DMPs. Annotation track indicates diagnosis group (control, salmon; FXTAS, teal). Hierarchical clustering was performed using one-minus Spearman correlation as the distance metric. **(B)** Methylation effect-size and significance plots across all tested CpG probes. The left panel shows mean methylation beta value in the baseline (*β*_Control, *x*-axis) against the methylation difference between FXTAS cases and controls (*Δβ* = *β*_FXTAS − *β*_Control, *y*-axis). The right panel shows *Δβ* against –log_10_(FDR-adjusted *p* value). Significant DMPs (|*Δβ*| > 0.10 and FDR < 0.05) are highlighted in red; dashed lines indicate the corresponding effect-size and significance thresholds. **(D)** Distribution of DMPs according to the number of genes to which they were annotated. Twenty-two DMPs were not assigned to a gene, 65 were assigned to one gene, nine to two genes, and two to three genes. **(E)** Distribution of DMP-associated genes according to the number of DMPs mapped to each gene. Sixty-two genes harbor a single DMP, 8 genes harbor 2–7 DMPs. **(F)** Distribution of the number of all-probes and differentially methylated probes (DMP) in different CpG regions. The left plot shows the number of probes while the right one shows the percentage. **(G)** Distribution of all tested probes and DMPs across gene features. The left panel shows probe counts, and the right panel shows the corresponding percentages. Exon-intron and intron-exon designate the 200-bp regions flanking the corresponding boundaries.

#### 3.2.1 Differentially methylated probes (DMP) and differentially methylated regions (DMR)

Applying thresholds of |Δ*β*| > 0.10 and FDR-adjusted *p*-value < 0.05, we identified 98 significant probes, 48 hypermethylated (Δ*β* range +0.100 to +0.241) and 50 hypomethylated (Δ*β* range −0.100 to −0.229) probes in FXTAS relative to controls (Figure 1B). The slight predominance of hypomethylation (51% of 98 CpG DMPs) did not reach statistical significance (binomial test, H_0_ = 0.5, *p* = 0.92). Mean absolute effect sizes were similar between hypermethylated (mean |Δ*β*| = 0.134) and hypomethylated (mean |Δ*β*| = 0.140) probes.

Hierarchical clustering of the top DMPs in a heatmap confirmed partial separation of cases and controls (Figure 1C). A total of 98 CpG DMPs were identified: 22 mapped to no annotated gene, 65 to a single gene, nine to two genes, and two to three genes (Figure 1D). The DMPs were annotated to 70 genes (Figure 1E). Most genes (62/70) were tagged by a single differentially methylated probe, while three genes (*ENSG00000285799*, *NKAIN1*, and *RRM2P2*) carried two probes each, three genes (*CYP2E1, ENSG00000278518,* and *LINC02116*) carried three, one gene (*ENSG00000228404*) carried five, and one gene (*SPATC1L*) carried seven. All DMPs within each multi-probe gene were directionally consistent, providing increased confidence in these associations. Selected significant DMPs with their biological context are presented in Table 2, and the list of all 98 DMPs is provided in Supplementary Table S1.

**Table 2:** Selected significant differentially methylated CpG probes (DMP; |Δ*β*| > 0.10 and FDR < 0.05) and their annotated genes.

Probe ID	Gene	Δ*β*	FDR adj. p	Direction	Biological function
cg06460587	*LY6G5C*	+0.180	0.006	Hyper	MHC class III–region Ly6-family gene (immune-associated, function uncharacterized)
cg07319276	*TRAF3*	+0.144	0.002	Hyper	Negative regulator of non-canonical NF-κB/type I IFN signalling
cg18924562	*CLEC1A*	+0.120	0.002	Hyper	Inhibitory C-type lectin innate immune receptor
cg24679453	*PRDM16*	+0.109	0.001	Hyper	PR/SET-domain histone methyltransferase; neural stem-cell regulator
cg19408572	*DGKQ*	+0.125	0.031	Hyper	Diacylglycerol kinase-θ; synaptic vesicle recycling (4p16.3 PD locus)
cg21758314	*CDC42BPB*	+0.132	0.045	Hyper	MRCK*β* kinase; Rho-GTPase/cytoskeleton, neurite outgrowth
cg17448987	*MSI2*	+0.104	0.036	Hyper	Neural RNA-binding protein (Musashi-2)
cg17146406	*RCOR1*	+0.105	0.025	Hyper	CoREST corepressor complex (LSD1/HDAC); neuronal gene repression
cg03309253	*LTBP4*	+0.115	0.021	Hyper	Latent TGF-*β* binding protein; controls bioavailability of the immunomodulatory cytokine TGF-*β*
cg25250968	*NEDD9*	+0.103	0.047	Hyper	HEF1 scaffold; lymphocyte adhesion/migration; AD/PD susceptibility locus
cg05194426	*CYP2E1*	−0.153	0.008	Hypo	Cytochrome P450; oxidative stress/ROS, DHA epoxygenation (3 DMPs, all hypomethylated; most significant shown)
cg06103394	*PAQR4*	−0.205	0.013	Hypo	Membrane signalling protein; NRF2 stabilisation (oxidative stress)
cg13012494	*SPATC1L*	−0.210	0.022	Hypo	Spermatogenesis/cytoskeleton; 7 DMPs, mixed direction (promoter hypo/CDS hyper)
cg14162034	*LINGO1*	−0.140	0.031	Hypo	Nogo-receptor co-receptor; myelination inhibitor; essential-tremor risk gene
cg23279522	*MEIS1*	−0.153	0.017	Hypo	Myeloid/neurodevelopmental TALE transcription factor
cg13957186	*RHBDF2*	−0.113	0.014	Hypo	iRhom2; ADAM17-mediated TNF-α shedding, inflammatory signalling
cg22050426	*ELMO3*	−0.112	0.008	Hypo	ELMO–DOCK–Rac module; phagocytosis/engulfment
cg18936040	*NKAIN1*	−0.103	0.012	Hypo	Neuronal Na^+^/K^+^-ATPase–interacting protein
cg12993715	*ZIC1*	−0.117	0.039	Hypo	Cerebellar zinc-finger transcription factor
cg25631352	*ADD2*	−0.112	0.049	Hypo	*β*-adducin; synapse stability/plasticity
cg14228592	*SLC39A4*	−0.102	0.029	Hypo	Zinc importer (ZIP4)
cg01528321	*TSPAN14*	−0.104	0.026	Hypo	ADAM10 regulator; microglial; Alzheimer’s GWAS gene
cg16203592	*TMEM154*	−0.188	0.002	Hypo	Immune-cell–expressed transmembrane protein (lentivirus host factor); function uncharacterized
cg02160836	*MIR548AA2*	−0.117	0.002	Hypo	miR-548 family microRNA; suppresses type I/III interferon (antiviral) responses

*β* = mean *β*-value difference (FXTAS − control); FDR adj. *p* = Benjamini–Hochberg adjusted P (*β*-value model). Red shading/Hyper: hypermethylated; blue shading/Hypo: hypomethylated. The list of all 98 significant DMPs is provided in Supplementary Table S1.

We annotated all 98 DMPs with respect to gene-region and CpG-island context (Figures 1F,G). Compared with the background distribution of all probes included in the analysis, DMPs showed a greater proportion of probes located within CpG islands (35% of DMPs vs. 19% of all probes) and shores (27% vs. 18%; islands and shores combined, 62% vs. 37%), a modestly lower proportion of promoter-associated probes (32% vs. 38%), and a slightly higher proportion of intergenic probes (20% vs. 11%). Despite this overall relative depletion of DMPs at promoters, several of the loci central to our interpretation were located within CpG islands or their flanking shores. The *TRAF3* DMP lies within a CpG island, all *CYP2E1* DMPs lie within either a CpG island or an S-shore, and the *LINGO1* DMP is also CpG-island associated.

Region-level analysis identified 101 candidate DMRs (Supplementary Table S2). However, the large majority of these regions (93 of 101) were supported by only a single CpG probe, limiting their interpretation as robust region-level methylation changes. Only a small number of regions were supported by multiple probes, including three DMRs supported by two CpG probes. The DMR analysis therefore provided limited additional information beyond the DMP analysis.

Because *β* values are bounded and heteroscedastic, we repeated the complete differential methylation analysis using M-values (Du et al., 2010). The results were highly concordant between the two approaches. Across all tested CpG sites, effect sizes and adjusted *p*-values from the *β*-value and M-value analyses were strongly correlated (Pearson r = 0.98 for effect size and 0.95 for adjusted *p*-value), and linear regression of the two analyses yielded slopes of 0.95 (effect size) and 0.83 (adjusted *p*-value), with intercepts of 0.00037 and 0.016, respectively, indicating close agreement overall, although adjusted *p*-values tended to be somewhat smaller in the *β*-value analysis (Supplementary Figure S2). The numbers of DMPs identified by the two approaches were comparable, although each approach yielded some unique DMPs. DMPs unique to the *β*-value approach tended to have larger effect sizes, whereas adjusted *p*-values were highly similar. Because effect sizes (Δ*β*) are directly estimated by the model in the *β*-value approach and are more intuitively interpretable, we retained *β* values for the primary analysis and presentation, while the M-value analysis provides an additional statistical robustness evaluation.

#### 3.2.2 FMR1 locus methylation

Three CpG sites at the *FMR1* locus showed FDR-adjusted *p* < 0.05 when considered without effect size threshold. However, all three had |Δ*β*| values below the 0.10 effect size threshold (range 0.035–0.054) and therefore did not meet the primary definition of a DMP. Thus, no large-effect methylation changes were detected at the *FMR1* locus in FXTAS. This pattern is consistent with the premutation state, in which the *FMR1* promoter generally remains unmethylated, in contrast to the promoter hypermethylation characteristic of the full mutation in fragile X syndrome (FXS).

#### 3.2.3 DMP-associated candidate genes

Beyond the *FMR1* locus, several DMP-associated genes fell into biologically interpretable themes (Table 2), and their mechanistic implications are considered in the Discussion. *CYP2E1*, encoding a cytochrome P450 enzyme that is a major enzymatic source of reactive oxygen species in neurons and astrocytes (Shah et al., 2021), carried three CpG probes, all hypomethylated in FXTAS (mean Δ*β* = −0.16, range −0.21 to −0.15), located within a CpG island (one probe) or its south shore (two probes). Several DMPs mapped to neuronal and synaptic genes, including *LINGO1*, a negative regulator of myelination and oligodendrocyte differentiation (Mi et al., 2005) (Δ*β* = −0.141, CpG-island associated); *ADD2* (*β*-adducin; Δ*β* = −0.112 promoter and within a CpG island), *DGKQ* (Δ*β* = +0.1248, promoter and within a CpG island). Additional neuronal or synaptic genes included *NKAIN1, NEDD9, MSI2, MEIS1, SORD* and *TSPAN14*. Several DMP-associated genes encoded chromatin or epigenetic regulators, including *PRDM16* (Δ*β* = +0.109, intron), *RCOR1* (Δ*β* = +0.105, 1–5 kb upstream), *TADA2A* (Δ*β* = +0.103, 1–5 kb upstream), and *PRDM7* (Δ*β* = −0.125; intron). Additional transcriptional and chromatin-associated regulators included *RPRD2, TCF25, ESRRB, FOXM1*, and *TBX20*.

Several DMP-associated genes also have established roles in innate immune and NF-κB signaling. These included *TRAF3* (Δ*β* = +0.144, intron), a regulator of non-canonical NF-κB and type I interferon responses (Häcker et al., 2011); and *CLEC1A* (Δ*β* = +0.120, intron), a C-type lectin innate immune receptor. In contrast, several immune-related genes were hypomethylated, including the myeloid-lineage transcription factor *MEIS1* (Δ*β* = −0.153, 1–5 kb upstream); *RHBDF2*/iRhom2 (Δ*β* = −0.113, promoter), which regulates ADAM17-mediated TNF-α shedding and inflammatory signaling, and the phagocytosis regulator *ELMO3* (Δ*β* = −0.112, promoter). Additional immune-related loci included *LY6G5C* and *TSPAN14*. Finally, the zinc importer *SLC39A4* (Δ*β* = −0.102; promoter/CpG island) and the cerebellar zinc-finger transcription factor ZIC1 (Δ*β* = −0.117, intron) were hypomethylated.

### 3.3 Analysis of gene pathways, transcription-factor motifs, and protein–protein interaction networks

#### 3.3.1 Reactome pathway analysis

Approximately one-third of the submitted genes, including unannotated *ENSG* identifiers, pseudogenes, and long non-coding RNAs, had no Reactome pathway annotation and were therefore excluded from analysis. Among the genes that mapped to Reactome, 10 pathways reached nominal significance (*p* < 0.05, uncorrected; Table 3), but none remained significant after Benjamini-Hochberg correction for multiple testing (FDR ≥ 0.69 across repeated independent submissions of this gene set). Most of the nominally significant pathways were driven by a single overlapping gene, including “CYP2E1 Reactions”; “Zinc transporters”; and “Zinc influx into cells by the SLC39 gene family”.

**Table 3:** Reactome exploratory pathway analysis of 70 DMP-annotated genes.

Reactome pathway	Nominal *p*-value	Overlapping gene(s)	Biological theme
Biosynthesis of maresin-like SPMs	0.012	multiple	Inflammation-related/pro-resolving signaling
N-glycan trimming/elongation, cis-Golgi	0.010	multiple	Glycoprotein processing
G2/M DNA replication checkpoint	0.010	*FOXM1*	Cell cycle/DNA integrity
Biosynthesis of maresins	0.016	multiple	Inflammation-related/pro-resolving signalling
Axonal growth inhibition (RHOA activation)	0.018	*LINGO1* + 1 other	Axonal/neuronal function
CD163 mediating an anti-inflammatory response	0.018	multiple	Inflammation-related/pro-resolving signalling
Zinc influx via the SLC39 gene family	0.020	*SLC39A4* only	Zinc homeostasis
p75NTR regulates axonogenesis	0.020	*LINGO1* only	Axonal/neuronal function
CYP2E1 reactions	0.022	*CYP2E1* only	Oxidative stress/metabolism
Zinc transporters	0.030	*SLC39A4* only	Zinc homeostasis

Values shown are nominal, uncorrected *p*-values. Pathways are grouped by biological themes.

Although not significant after multiple-testing correction, these nominal associations grouped the DMP-associated genes into several biologically interpretable themes: (1) inflammation-related and pro-resolving mediators (SPMs, *p* = 0.012), biosynthesis of maresins (*p* = 0.016), and CD163-mediated anti-inflammatory response (*p* = 0.018); (2) axonal and neuronal function, including axonal growth inhibition via RHOA activation (*p* = 0.018) and p75NTR-regulated axonogenesis (*p* = 0.020); (3) oxidative stress and metabolism, represented by CYP2E1 reactions (*p* = 0.022); (4) zinc homeostasis, including zinc influx via the SLC39 family (*p* = 0.020) and zinc transporters (*p* = 0.030); (5) glycoprotein processing, represented by N-glycan trimming and elongation in the cis-Golgi (*p* = 0.010); and (6) DNA integrity, represented by the G2/M DNA replication checkpoint (*p* = 0.010).

#### 3.3.2 Cross-validation across additional functional annotation databases

To assess whether the exploratory Reactome findings were supported by independent functional annotation resources, we analyzed the 70 DMP-associated genes using g:Profiler (Supplementary Table S3). After g:SCS multiple-testing correction, 31 terms remained significant (adjusted *p* < 0.05), comprising 21 transcription-factor binding motif gene-sets, 9 miRNA target sets, and one Gene Ontology molecular-function term (enzyme binding). Among all significant g:Profiler results, the strongest enrichments were transcription-factor-motif associated gene-sets, including *ZNF432* (*p* = 0.005; 33 genes), *SALL2* (*p* = 0.005; 39 genes), *ZNF692*, and *KLF*-family signatures. Among the enriched miRNA target sets was miR-342, which has been implicated in TNF-α-induced microglial activation, NF-κB signaling, and neurotoxicity (Brás et al., 2020).

#### 3.3.3 Transcription-factor binding-site enrichment at DMP loci

Because the transcription-factor annotations identified by g:Profiler are based on gene-set membership rather than direct analysis of DNA sequence, we independently tested for transcription-factor binding-site enrichment in the genomic regions surrounding the 98 DMPs. Separate sequence-based motif enrichment analyses were performed using the HOMER and JASPAR vertebrate motif libraries, examining ±100-bp regions centered on each DMP and using array-probe-derived background regions.

Using HOMER, none of the 414 known motifs tested remained significant after BH-correction for multiple testing (minimum *q* = 0.34; Supplementary Table S4). The top-ranked motif, AMYB (HTH family), was present in 26 of 98 DMP-centered sequences (26.5%) compared with 14.0% of background sequences (nominal *P* ≈ 1 × 10^−3^, *q* = 0.34). Five additional motifs had nominal *p* < 0.05 (MYB, ATF1, SPDEF, JUN/AP-1, and BACH2) but none survived multiple-testing correction (q ≥ 0.75). Several of the nominally enriched motifs belonged to the bZIP/AP-1/CREB family (ATF1, JUN/AP-1, BACH2, ATF7, FOSL2).

We next repeated the analysis using the JASPAR vertebrate CORE motif collection with the same 98 DMP-centered regions and array-probe-derived background. Again, no motif remained significant after Benjamini–Hochberg correction (minimum q = 0.16 for HOXD13 and ZNF780B; all remaining motifs q ≥ 0.74). Among the nominally top-ranked motifs, several belonged to the AP-1/bZIP family (BACH1, JUNB, FOSL1, FOS:JUND, FOSL2, ATF2), broadly paralleling the pattern observed using the HOMER library. Although these motif enrichments did not survive multiple-testing correction, the recurrent ranking of AP-1/bZIP family motifs across both sequence-based analyses provides a hypothesis for targeted follow-up in the context of the innate immune and NF-κB-related signals identified in the other analyses.

#### 3.3.4 Protein–protein interaction (PPI) network analysis

STRING v.12 analysis of the DMP-associated genes yielded a sparse PPI network. The majority of the ~62 genes represented in STRING were unconnected singletons, with only five pairwise interactions identified, which included TRAF3–CDC42BPB, MEIS1–TBX20, PRDM16–RCOR1, PAQR4–PRSS21, and PRSS21–SPATC1L. Most interactions were of low-to-medium confidence (combined score 0.40–0.66) and were driven primarily by automated text-mining evidence. The PRDM16–RCOR1 interaction showed the strongest experimental support (experimental sub-score 0.43), consistent with the chromatin/epigenetic-regulator theme seen in the functional annotation analyses. The immune regulator TRAF3 was connected only to the cytoskeletal kinase CDC42BPB through a text-mining-supported edge. Overall, the STRING analysis did not identify a coherent PPI module corresponding to the innate-immune/NF-κB signal observed at the methylation and gene-annotation levels (Supplementary Figure S3).

### 3.4 Overlap analysis with an FXTAS mouse transcriptomic dataset and brain methylation signatures from other neurodegenerative disorders

We compared the DMP-associated genes identified in our study with differentially expressed genes reported in a recent mouse multi-omic study of FXTAS (Jin et al., 2026). This comparison spanned species, brain regions, and molecular layers; our data represent DNA-methylation changes in human postmortem prefrontal cortex, whereas the comparison dataset was derived from bulk RNA-seq of cortex, cerebellum, and hippocampus from a CGG-repeat–expansion mouse expressing 99 CGG repeats brain-wide through Nestin-Cre. Seven of our DMP-associated genes were also reported as differentially expressed in that study: *DGKQ, ELMO3, LRIG1, LTBP4, MEIS1, PAQR4*, and *ZIC1*. Notably, all seven were reported as direct hnRNPA2B1 targets by eCLIP and showed altered expression in FXTAS-model GABAergic neurons, including in cortex (*DGKQ, ELMO3, LTBP4, PAQR4*) and/or cerebellum (*DGKQ, ELMO3, LRIG1, MEIS1, ZIC1*). Four of these genes have functions relevant to immune or stress-response pathways. *ELMO3* drives phagocytosis/engulfment, a central microglial innate-immune function, *MEIS1* is a myeloid-lineage transcription factor, *LTBP4* regulates the bioavailability of TGF-*β*, a major immunomodulatory/anti-inflammatory and pro-fibrotic cytokine, and *PAQR4* has been linked to immune signaling and oxidative-stress responses through NRF2. Methylation and expression directions were concordant for 4/7 overlapping genes, including promoter-hypermethylated and downregulated *DGKQ* and *LTBP4*, and discordant for three (Supplementary Table S5). Given the differences in species, brain region, cell context, and molecular modality, this overlap is best interpreted as convergent dysregulation of these loci rather than evidence for a direct cis-regulatory relationship between DNA methylation and gene expression.

Next, we compared the DMP-associated genes with published brain methylation signatures from Parkinson’s disease (PD; Harvey et al., 2025); Alzheimer’s disease (AD; Smith et al., 2021); and Huntington’s disease (HD; Wheildon et al., 2025). No DMP-associated genes overlapped between the FXTAS gene set and the AD, PD, or HD methylation signatures examined.

### 3.5 Proteomic analysis reveals suppression of inflammatory signaling proteins in FXTAS

#### 3.5.1 Profiling of FXTAS cortex using an Olink targeted inflammatory panel

Motivated by the enrichment of innate immune/NF-κB signals in the methylation analysis, we sought validation at the protein level by profiling the same prefrontal cortex region with the Olink Inflammation panel in 9 male FXTAS cases and 12 male controls. As with the methylation analysis, surrogate variable analysis (SVA) was applied. Two surrogate variables were retained, and after SVA adjustment, principal component analysis of the inflammation proteome showed partial separation between FXTAS and control samples (Figure 2A).

**Figure 2:**
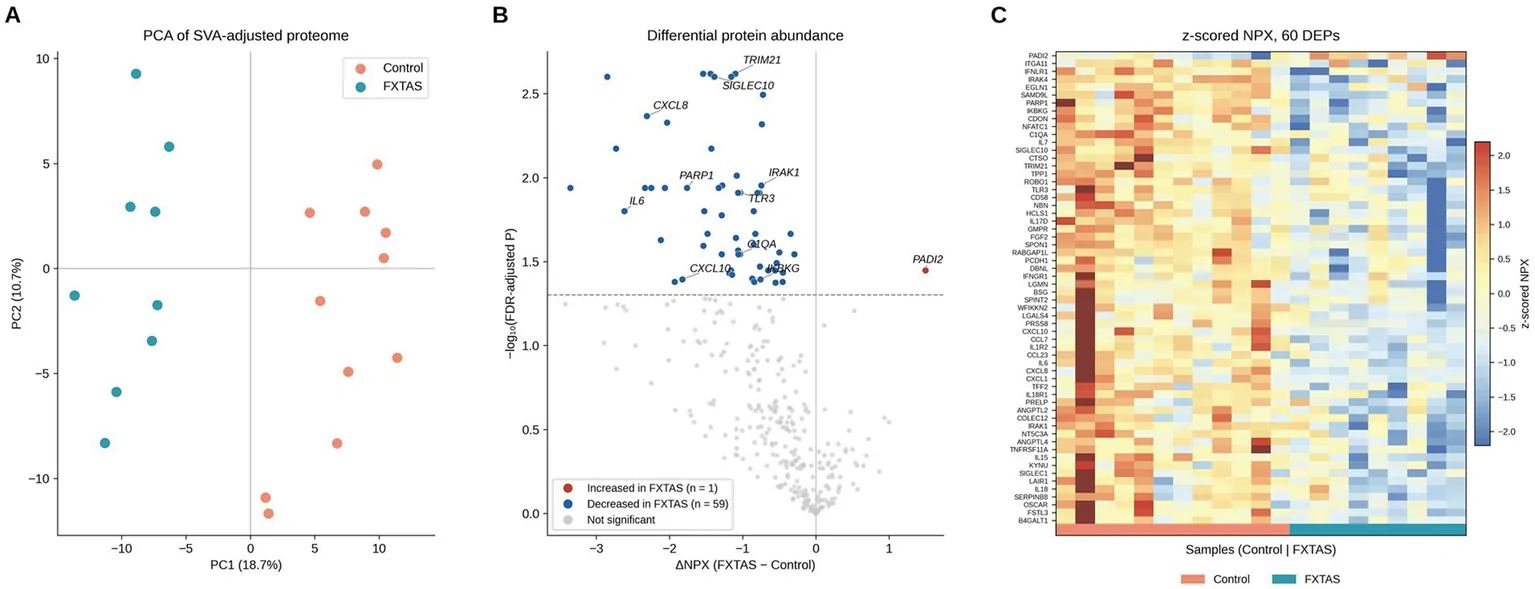
Targeted proteomic analysis of inflammation-related proteins in FXTAS prefrontal cortex. Proteins were measured using the Olink Inflammation panel, and analyses were adjusted using SVA (9 FXTAS and 12 controls). **(A)** PCA of SVA-adjusted normalized protein expression (NPX) values. FXTAS cases are shown in teal and controls in salmon. *PC1* and *PC2* explain 18.7 and 10.7% of the variance, respectively. **(B)** Volcano plot of differential protein abundance. The x-axis shows the SVA-adjusted NPX difference between FXTAS and controls (Δ*NPX* = FXTAS − control), and the y-axis shows –log_10_(FDR-adjusted *p* value). Proteins significantly increased and decreased in FXTAS are shown in red and blue, respectively; nonsignificant proteins are shown in gray. The horizontal dashed line denotes FDR = 0.05. **(C)** Heatmap of *z*-scored, SVA-adjusted NPX values for the significantly altered proteins. Columns represent samples and rows represent proteins. Protein labels in blue are FXTAS. Rows are clustered hierarchically. The sample annotation track indicates diagnostic group (control, salmon; FXTAS, teal); the heatmap scale indicates relative protein abundance (higher, red; lower, blue). Proteins were hierarchically clustered.

Differential abundance analysis identified 60 proteins significantly altered in FXTAS compared with controls (FDR < 0.05), of which 59 showed lower abundance and one showed higher abundance in FXTAS (Figure 2B; Table 4). The proteins with decreased abundance included several functionally related groups, notably pro-inflammatory chemokines (CXCL8/IL-8, CXCL10/IP-10, CXCL1, CCL7, CCL23), interleukins and their receptors (IL6, IL7, IL15, IL17D, IL18, IL1R2, IL18R1), innate immune and NF-κB signaling components (TLR3, IRAK1, IRAK4, IKBKG/NEMO, TRIM21), the classical complement component C1QA (Stevens et al., 2007), microglial receptors (SIGLEC1, SIGLEC10), and the neurotrophic factor FGF2. The largest individual effects were observed for LGALS4 (galectin-4, ΔNPX = −3.36) and PARP1 (−1.76), while the most statistically significant changes included TRIM21, FGF2, and CXCL8.

**Table 4:** Selected differentially abundant Olink inflammation proteins in FXTAS prefrontal cortex.

Protein	ΔNPX	FDR	Biological function	Holm et al. (2020)
IL7	−1.44	0.002	Cytokine	–
TRIM21	−1.10	0.002	Innate immune E3 ligase	–
CCL7	−2.85	0.002	Chemokine (MCP-3)	–
FGF2	−1.16	0.002	Growth factor	–
SIGLEC10	−1.39	0.002	Microglial receptor	–
CXCL8	−2.31	0.004	Chemokine (IL-8)	–
IL1R2	−2.73	0.007	Cytokine receptor (IL-1R2)	–
IRAK1	−0.75	0.011	NF-κB signaling	Concordant
IL15	−1.28	0.011	Cytokine	–
PARP1	−1.76	0.012	DNA damage/inflammation	Concordant
IL17D	−1.33	0.012	Cytokine	–
TLR3	−1.04	0.012	Innate immune (TLR)	–
IL6	−2.62	0.016	Cytokine	–
SIGLEC1	−1.48	0.022	Microglial receptor	–
CXCL1	−2.12	0.024	Chemokine (GRO-α)	–
IL18R1	−1.54	0.025	Cytokine receptor (IL-18R1)	–
C1QA	−1.06	0.029	Complement	–
CCL23	−1.16	0.036	Chemokine (MIP-3)	–
IL18	−1.15	0.038	Cytokine	–
CXCL10	−1.83	0.040	Chemokine (IP-10)	–
IRAK4	−0.85	0.042	NF-κB signaling	–
IKBKG	−0.76	0.040	NF-κB signaling (NEMO)	Concordant
KYNU	−1.08	0.010	Kynurenine pathway	–

Of the 60 proteins significantly altered in FXTAS (Olink inflammation panel, SVA-adjusted, FDR < 0.05), 23 are shown here, selected based on the magnitude of the abundance difference and biological relevance to FXTAS. Negative ΔNPX indicates lower abundance in FXTAS compared to controls. Directional concordance with the mass spectrometry-based FXTAS cortex proteome reported by Holm et al. (2020) is also indicated. Biological functions were manually annotated based on the published literature. The complete list of 60 differentially abundant proteins is provided in Supplementary Table S6.

Because 59 of the 60 significantly altered proteins showed lower abundance in FXTAS, we examined whether this pronounced directional imbalance could reflect differences in sequencing depth or a systematic shift introduced during normalization (Supplementary Figure S4). Total sequencing depth did not differ significantly between FXTAS and control samples (*p* = 0.24; Supplementary Figure S4A). A lower median signal for biological assays was already evident in the raw, pre-normalization counts in FXTAS, although the between-group difference in sample-level medians was not significant (*p* = 0.43; Supplementary Figures S4B,D). In contrast, the Olink spike-in controls showed a small shift in the opposite direction (*p* = 0.033; Supplementary Figure S4C). Importantly, the magnitude of the FXTAS–control difference across biological assays was nearly unchanged after normalization (Δ = −0.32 log_2_ units in raw counts versus Δ = −0.30 NPX; Supplementary Figure S4E). Together, these analyses indicate that the near-uniform reduction in inflammatory protein abundance is unlikely to be explained solely by sequencing depth, internal-control behavior, or NPX normalization.

#### 3.5.2 Concordance with a published FXTAS cortex proteome

We next compared our findings to a previous proteomic study of FXTAS postmortem cortex based on untargeted mass spectrometry (MS) analysis of 8 FXTAS and 6 control male cortices from the same UC Davis brain repository (Holm et al., 2020). That study independently reported reduced abundance of inflammation-associated proteins in FXTAS cortex, consistent with the overall direction seen in our Olink analysis. Direct protein-level comparison was limited by differences in platform coverage, i.e., of the 60 proteins significantly altered in our targeted analysis, only 19 were detected in the MS dataset. This limited overlap likely reflects, at least in part, the enrichment of the Olink panel for low-abundance cytokines and chemokines, including CXCL8, CXCL10, IL6, and IL15, which are challenging to detect by untargeted MS.

Among the 19 proteins measured by both platforms, 13 (68%) showed concordant directionality between studies, including the NF-κB-associated proteins IRAK1, IKBKG/NEMO, and PARP1, as well as the axon-guidance protein SPON1, which showed reduced abundance in both datasets and was near significance in the published study. The correlation of quantitative effect sizes among co-measured proteins was low (Pearson *r* = 0.20), as expected, given the non-overlapping protein coverage and the modest cohort sizes. Thus, the two studies converge on the broader biological finding of reduced abundance of inflammation-associated proteins in FXTAS cortex across distinct proteomics approaches, while not constituting direct protein-by-protein replication.

Together, the proteomic findings provide cross-omic support for dysregulation of innate immune and NF-κB related pathways identified in the methylation analysis. *TRAF3*, a regulator of non-canonical NF-κB and type I interferon signaling, contained one of the most significant methylation signals in the study. Independently, the proteomic data showed reduced abundance of multiple components of innate immune signaling, including TLR3, IRAK1, IRAK4, and IKBKG/NEMO, together with broad decreases in inflammatory effectors. Although these proteins participate in distinct branches of innate immune signaling rather than a single linear pathway, their coordinated alteration provides convergent evidence for disruption of inflammatory signaling in FXTAS cortex.

## 4 Discussion

This study presents the first genome-wide DNA methylation analysis of postmortem prefrontal cortex tissue from individuals with FXTAS. Profiling 721,802 CpG sites across 27 male FXTAS cases and 29 male controls, we identified 98 significant differentially methylated positions (DMPs), comprising 48 hypermethylated and 50 hypomethylated sites that mapped to 70 annotated genes. Genes associated with these methylation changes implicated several biological processes relevant to FXTAS, including oxidative stress, axonal integrity, zinc homeostasis, and most notably, given independent support at the protein level, innate immune/NF-κB signaling. Together, these findings identify discrete epigenetic alterations in the FXTAS prefrontal cortex and highlight several mechanistic hypotheses for future functional investigation.

### 4.1 Convergence of epigenetic dysregulation on CGG-repeat RNA toxicity

This study showed that no large-scale methylation changes were detected at the *FMR1* locus. Beyond *FMR1*, however, DMP-associated genes converged on several biological themes including oxidative stress, innate immune/NF-κB signaling, myelination, zinc homeostasis, and chromatin/non-coding RNA regulation, which we frame here as testable links to CGG-repeat RNA toxicity. Expanded CGG-repeat RNA is known to sequester RNA-binding and chromatin-associated proteins. If such sequestration also affects DNA or histone methyltransferases, it could lead to locus-specific hypomethylation at genes normally maintained in a repressed state by these enzymes.

*CYP2E1* was among the most internally consistent single-gene findings. *CYP2E1* is a major enzymatic source of reactive oxygen species in brain (Shah et al., 2021), and oxidative stress and mitochondrial dysfunction are established features of FXTAS (Ross-Inta et al., 2010). CYP2E1-derived ROS also contributes to dopaminergic vulnerability in Parkinson’s disease (Shah et al., 2021; Houldsworth, 2024), and CYP2E1 metabolizes the DHA-derived precursors of maresins and other specialized pro-resolving mediators that promote resolution of neuroinflammation (Serhan et al., 2009; Serhan et al., 2015). We therefore raise the hypothesis that *CYP2E1* hypomethylation, if associated with increased expression, could alter DHA metabolism and impair pro-resolving inflammatory signaling, a process reported to be disrupted in the Alzheimer’s disease brain (Zhu et al., 2016). Independently, *CYP2E1*-derived ROS is an established activator of NF-κB signaling, (Morgan and Liu, 2011) providing a potential mechanistic link between the oxidative-stress and innate-immune signatures observed here. The transcriptional and functional consequences of *CYP2E1* hypomethylation remain to be established and could be tested by assessing *CYP2E1* RNA and protein expression, together with ROS production.

*TRAF3* carried one of the more significant DMPs identified in this study. TRAF3 is a negative regulator of non-canonical NF-κB and type I interferon signaling (Häcker et al., 2011). Thus, its hypermethylation, if associated with reduced expression, could diminish an important brake on inflammatory signalling. More broadly, DMPs occurred in several genes encoding regulators of innate immunity and NF-κB signaling, with an intriguing directional pattern. Specifically, negative regulators of inflammatory signaling, including *TRAF3* (Häcker et al., 2011) and CLEC1A (Lopez Robles et al., 2017), were hypermethylated, whereas genes involved in myeloid-cell activation, phagocytic function, and inflammatory effector pathways, including RHBDF2/iRhom2 (Adrain et al., 2012), MEIS1, and ELMO3, were hypomethylated.

The Olink proteomic dataset converged on related pathways and included altered abundance of the NF-κB pathway components TLR3, IRAK1, IRAK4, and IKBKG/NEMO, as well as complement C1QA and multiple cytokines and chemokines. Rather than interpreting the predominantly decreased inflammatory protein abundance as evidence for an absence of inflammation, we hypothesize that the combined epigenetic and proteomic findings may reflect a chronically engaged but dysregulated or inadequately resolved immune state. This interpretation is consistent with immune dysregulation observed in isolated PBMCs from female *FMR1* premutation carriers without FXTAS (Careaga et al., 2014) and with glial abnormalities described in FXTAS brain (Dias et al., 2023; Dufour et al., 2024). Importantly, the reduced inflammatory protein signature observed here in prefrontal cortex may not reflect a uniform response across the FXTAS brain. Elevated pro-inflammatory cytokines, including IL-12 and TNF-α, have been reported in the cerebellum (Dufour et al., 2021), suggesting that inflammatory changes may differ across affected brain regions. Notably, the reported cerebellar TNF-α elevation is directionally consistent with *RHBDF2*/iRhom2 hypomethylation, although whether this methylation change increases *RHBDF2* expression and ADAM17-mediated TNF-α shedding in FXTAS remains to be established.

White matter disease is a defining feature of FXTAS in males, and hypomethylation of *LINGO1* at a CpG-island-associated probe provides a potential link to oligodendrocyte and myelin biology. *LINGO1* inhibits oligodendrocyte differentiation, myelination, and axonal regeneration via RhoA signaling, is upregulated in demyelinating disease, and has been investigated as a therapeutic target (opicinumab) in multiple sclerosis (Mi et al., 2005). If *LINGO1* hypomethylation is associated with increased expression, it could contribute to impaired oligodendrocyte function and remyelination. Hypomethylation of *NKAIN1*, *ADD2*, and *COLQ* further suggests alteration of neuronal membrane and synaptic processes.

Altered methylation of genes involved in zinc biology was also notable. These included the zinc importer *SLC39A4* (ZIP4) and the cerebellar zinc-finger factor *ZIC1* (Grinberg et al., 2004). Zinc dysregulation has been linked to mitochondrial dysfunction and impaired antioxidant defense in neurodegenerative diseases, and FMRP, which is modestly reduced in FXTAS, regulates translation of zinc-transporter mRNAs (Lozano et al., 2016). In addition, altered methylation of chromatin regulators including *RCOR1* (CoREST) (Andrés et al., 1999), *TADA2A* and *PRDM7* points to epigenetic perturbations extending beyond the *FMR1* locus. Collectively, these observations suggest that CGG-repeat-associated toxicity may intersect with pathways involving oxidative stress, neuroinflammation, myelination, zinc homeostasis, and chromatin regulation, each of which represents a testable hypothesis for mechanistic follow-up.

Several DMP-associated genes also have established roles in other neurodegenerative conditions, raising the possibility of shared molecular mechanisms across late-onset brain disorders. *CYP2E1* hypomethylation links FXTAS with oxidative stress pathways implicated in Parkinson’s disease (Shah et al., 2021), while *RCOR1* (CoREST complex), hypermethylated in our dataset, encodes a transcriptional repressor whose disruption has been implicated in Huntington’s disease (Zuccato et al., 2003). *LINGO1* hypomethylation links FXTAS to pathways relevant to demyelinating disease (Mi et al., 2005), and *TRAF3*, a regulator of NF-κB signaling, participates in neuroinflammatory signaling common to multiple neurodegenerative conditions. Despite these functional parallels, the FXTAS DMP-associated gene set showed no detectable gene-level overlap with the AD, PD, or HD brain methylation signatures examined here. This lack of overlap should not be interpreted as demonstrating an FXTAS-specific epigenetic signature, given differences in brain regions, array platforms, cohort composition, and analytical thresholds across studies. Comparison with an independent multi-omic FXTAS mouse-model study (Jin et al., 2026) identified seven of our DMP-associated genes that were also differentially expressed in that study (*DGKQ, ELMO3, LRIG1, LTBP4, MEIS1, PAQR4, and ZIC1*). This limited convergence likely reflects, at least in part, the cross-species, cross-region, and cross-molecular nature of the comparison, together with the underlying EPIC array probe distribution, which includes a substantial proportion of non-promoter CpG sites.

### 4.2 Limitations

Several limitations should be considered when interpreting these findings. First, pathway, ontology term, or transcription-factor motif analyses provided primarily exploratory or nominal significance signals, and the protein–protein interaction network among DMP-associated genes was sparse. Second, DNA methylation was profiled from bulk prefrontal cortex tissue, which contains a heterogeneous mixture of neurons, astrocytes, oligodendrocytes, microglia, endothelial cells, and other cell populations. Single-nucleus transcriptomic data from an independent FXTAS-spectrum cohort (Dias et al., 2023) suggest that at least some relevant molecular changes, particularly glial inflammatory responses, may be cell-type-specific, which our bulk-tissue design cannot directly resolve. Although SVA was used to control for latent sources of variation, it cannot fully distinguish cell-type composition effects from disease-associated methylation changes.

Third, this is a cross-sectional study of end-stage postmortem FXTAS tissue and therefore cannot establish the temporal relationship between methylation changes and disease onset or progression. Fourth, quantitative neuropathological measures such as density of ubiquitin-positive intranuclear inclusions or white-matter lesion burden, and detailed clinical severity or disease-duration measures were not available for this cohort, so we could not assess relationships between methylation or protein changes and disease severity. Fifth, we did not identify a publicly available genome-wide methylation dataset from FXS postmortem brain with matched controls, preventing direct comparison of the FXTAS profile to the methylation changes associated with the *FMR1* full mutation in FXS. Functional validation of the proposed epigenetic mechanisms will therefore be an important next step.

With respect to the proteomic analysis specifically, additional limitations apply. Postmortem brain tissue is susceptible to variation related to tissue handling, agonal state, and protein preservation across brain bank collections. We sought to mitigate these effects by applying SVA to account for latent variation, and by benchmarking the findings against an independent FXTAS cortex proteomic dataset, although residual confounding cannot be excluded. The Olink cohort was also smaller than the methylation cohort, limiting statistical power, and profiling was restricted to an inflammation panel rather than an unbiased proteome-wide assay. Accordingly, these results reflect the inflammation-related proteome rather than the full spectrum of protein alterations in FXTAS. NPX values represent relative rather than absolute protein quantification. In addition, the methylation and proteomic analyses were performed on overlapping but not identical sample sets, precluding formal sample-level integration of the two molecular layers.

Lastly, the DNA methylation changes measured by the EPIC array cannot be assumed to directly correspond to changes in RNA expression or protein abundance. Many EPIC probes are located in genomic regions without a simple or established relationship to transcription, and DMPs identified here were relatively depleted from promoters and enriched in intergenic regions. A straightforward silencing/de-repression model therefore should not be assumed for every locus. Moreover, even when methylation affects transcription, protein abundance is further shaped by translation, secretion, degradation, and cell-type composition. Consequently, strong one-to-one correspondence between individual methylation changes and inflammatory protein abundance would not necessarily be expected. Nevertheless, convergence of independently identified methylation and proteomic changes on related inflammatory pathways supports a potential role for altered immune signaling in FXTAS.

## 5 Conclusion

This study provides the first genome-wide DNA methylation map of FXTAS postmortem prefrontal cortex, identifying 98 significant CpG DMPs across 70 genes. These findings reveal epigenetic alterations beyond the *FMR1* locus and nominate candidate gene-level changes involving oxidative stress (*CYP2E1*), myelination (*LINGO1*), zinc homeostasis (*SLC39A4, ZIC1/4*), synaptic function (*SYT3*), chromatin regulation (*RCOR1, TADA2A*), and, most prominently in this dataset, innate immune/NF-κB signaling (*TRAF3, RCOR1, MSI2*). Hypermethylation of *TRAF3*, one of the more significant DMPs identified, together with reduced abundance of multiple innate immune and NF-κB-related proteins, including TLR3, IRAK1, IRAK4, and complement C1QA, supports convergent evidence for altered inflammatory signaling in FXTAS cortex. Among the findings of this study, the convergence between methylation changes in immune-regulatory genes and the predominantly reduced inflammatory protein signature represents a particularly notable observation warranting further investigation. Future studies integrating DNA methylation, transcriptomics, proteomics, and metabolomics in FXTAS brain tissue, together with longitudinal epigenome-wide studies in premutation carriers and direct functional validation of the candidate genes identified here, will be important for defining the biological significance of these findings and evaluating their potential relevance to biomarkers and therapeutic targets.

## Data Availability

The DNA methylation data generated in this study have been deposited in the NCBI Gene Expression Omnibus (GEO) and are accessible through GEO Series accession number GSE: GSE347467.

## Supplementary material

Supplementary Table S1. Complete list of the 98 significant cg-type DMPs (|Δβ| > 0.10, FDR < 0.05) with Δβ, FDR-adjusted p-value, gene annotation, gene feature, and CpG-island context.

Supplementary Table S2. Candidate differentially methylated regions (DMR, n = 101) with number of supporting probes and gene annotation.

Supplementary Table S3. Complete g: Profiler functional enrichment results for DMP-associated genes.

Supplementary Table S4. Complete ranked results from the HOMER and JASPER known-motif enrichment analyses of DMP regions.

Supplementary Table S5. DMP-associated genes overlapping with dysregulated hnRNPA2B1 targets in an FXTAS mouse model.

Supplementary Table S6. Full list of the 60 differentially abundant Olink inflammation proteins.

Supplementary Figure S1. Clinical and technical covariates and their relationships with surrogate variables in the FXTAS methylation dataset. Distributions of (A) age and (B) postmortem interval (PMI) are shown for FXTAS and controls using boxplots and density plots. (C) The correlation heatmap summarizes associations among the estimated surrogate variables (SVs) and known biological and technical covariates, including age, PMI, diagnosis, and EPIC array platform. Correlation values are shown by color intensity, with darker red indicating stronger associations.

Supplementary Figure S2. M-value sensitivity analysis. Comparison of effect sizes, adjusted *P*-values, and DMP overlap between the β-value and M-value differential methylation analyses. (A) Venn diagram showing the overlap of DMPs identified using the β-value and M-value approaches. Scatter plots comparing (B) effect sizes and (C) -log_10_(adjusted *P*-value) between the two approaches. DMPs are colored based on the classification shown in (A).

Supplementary Figure S3. STRING protein–protein interaction network of the DMP-associated genes. Nodes represent DMP-associated genes and edges represent protein–protein associations identified in STRING v12.0.

Supplementary Figure S4. Evaluation of potential technical sources of the global decrease in Olink inflammatory protein abundance in FXTAS. (A) Total sequencing depth per sample in FXTAS and control groups. (B) Distribution of sample-level median raw counts for biological assays before NPX normalization. (C) Sample-level median raw counts for the 12 internal Olink spike-in control assays. (D) Per-sample distributions of raw biological-assay counts, illustrating the overall downward shift in FXTAS before normalization. (E) Comparison of the FXTAS–control difference before and after NPX normalization, showing a similar magnitude of reduction in raw log₂ counts (Δ = −0.32) and normalized NPX values (Δ = −0.30).
